# The Renewal of Cancer Immunotherapy

**DOI:** 10.3390/vaccines11030592

**Published:** 2023-03-04

**Authors:** Jenny Bulgarelli, Sara Pignatta, Massimiliano Petrini, Laura Ridolfi

**Affiliations:** Immunotherapy, Cell Therapy and Biobank Unit, IRCCS Istituto Romagnolo per lo Studio dei Tumori (IRST) “Dino Amadori”, 47014 Meldola, Italy

Cancer immunotherapy embraces many current, promising therapeutic approaches to eradicate tumors by activating host antitumor activity. Immune checkpoint inhibitors (ICBs) and targeted therapies have dramatically improved outcomes in patients with cancer, but many patients do not benefit from them. To fill this therapeutic gap, there is an urgent need for basic and translational research that extends beyond well-designed clinical studies to identify new therapeutic combinations and mechanisms of action.

There are several immunotherapy approaches available, such as ICBs, anti-cancer vaccines, and adoptive immunotherapies, which can be used either alone or in combination to increase their efficacy and safety, and to treat or prevent metastatic disease [1].

Regarding immunotherapy combinations, randomized controlled, double-blind phase II CheckMate 069 and phase III CheckMate 067 trials established the combination of nivolumab and ipilimumab as a standard care option for patients with metastatic melanoma. These studies paved the way to substantial development in the efficacy of melanoma treatment versus the standard median survival of 8 months a decade ago [2,3].

Other papers provide insights into CAR-T cell immunotherapy (chimeric antigen receptor T cell immunotherapy). These engineered T cells can quickly identify and destroy target cells using their CAR receptors, and are accurate, flexible, spectral, and durable. CAR-T cells have demonstrated remarkable curative effects in the treatment of acute leukemia and non-Hodgkin lymphoma; moreover, researchers recently found that this cell therapy is effective in treating patients with glioblastoma and can remove 80% of a tumor. Additionally, it has been demonstrated that the combination of the anti-cancer vaccine and CAR-T therapy can stimulate the immune system to produce memory T cells and prevent the recurrence of tumors [4,5,6].

A recently published study by Linda M. Liau et al. reports the overall survival (OS) and safety outcomes of a phase 3 non-randomized controlled trial that tested an autologous tumor lysate-loaded dendritic cell vaccine (DCVax-L) combined with standard-of-care (SOC) for the treatment of glioblastoma [7]. Dendritic cells introduce tumor antigens to the immune system, prime T cells, and mobilize antitumor responses [8]. In the abovementioned study, the authors enrolled a total of 331 patients and found that adding DCVax-L to SOC was associated with a clinically meaningful and statistically significant improvement in the median OS of patients with both newly diagnosed (nGBM) and recurrent glioblastoma (rGBM). Additionally, they observed that patients who received DCVax-L survived for years after completing their vaccine doses, which could be due to an effective memory immune response [9].

Another promising option is adoptive immunotherapy. In particular, the use of tumor-infiltrating lymphocytes (TILs), first described in the 1980s by Rosenberg and colleagues [10], is a personalized treatment for cancer based on the infusion of autologous T lymphocytes that have been obtained directly from surgically removed autologous tumors, and then, expanded in culture via interleukin-2 stimulation.

In an outstanding study recently published in the *NEJM* by John B.A.G. Haanen and coworkers, a trial was conducted in which, for the first time, a TIL-based approach was directly compared to standard-of-care treatment (in this case, ipilimumab), with excellent results [11].

Upon exploring the possible mechanism by which TIL therapy operates in patients who have failed anti-PD-1 treatment, the authors observed that resistance mechanisms, in this case, are mostly delivered by the tumor microenvironment (TME). Indeed, when they removed these cells from their natural environment, reactivated them, increased their quantity to very large numbers, and returned them to the patients, they were able to overcome some escape mechanisms. Moreover, the observation that clinical benefit correlates with a higher frequency of cells that recognize tumor neoantigens has prompted efforts to enrich tumor-specific clones during culture; additionally, T cell-intrinsic characteristics such as “stemness” (or stem cell properties), persistence, and functionality play major roles in the effectiveness of this “live” immunotherapy. It is noteworthy that TIL has the potential to benefit patients with a wide range of solid tumors, and trials are currently underway for many cancer types, including lung, cervical, and head and neck cancers.

Additionally, while fewer randomized studies have been conducted in a neoadjuvant compared with an adjuvant setting, neoadjuvant treatment is becoming the preferred approach. It has been demonstrated that the neoadjuvant use of checkpoint inhibitors activates both the priming phase of immunity within the tumor tissue and the effector phase within the TME. This dual attack is less extensive if the macroscopic tumor has already been removed, as is the case for adjuvant therapy; moreover, the data show that neoadjuvant immunotherapy expands more T cell clones than adjuvant treatment in the periphery.

Recent results of a randomized phase II study of adjuvant therapy (AT) versus neoadjuvant therapy (NAT), using single-agent pembrolizumab (PEM) in a total of 313 high-risk resectable melanoma (SWOG S1801) patients, were presented by Patel S. et al. They found that NAT improves event-free survival (EFS) compared to AT in this cohort of patients [12].

Following a translational study of T cell dysfunction and exclusion signatures in tumors, Peng Jiang and co-workers recently presented important evidence regarding immunotherapy response prediction. The authors developed TIDE (Tumor Immune Dysfunction and Exclusion), a computational method of identifying the factors that underlie two mechanisms of tumor immune escape. They identified signatures of T cell dysfunction from large tumor cohorts by testing how the expression of each gene in tumors interacts with the CTL infiltration level to influence patient survival, and modeled factors that prevent T cell infiltration into tumors using expression signatures from immunosuppressive cells. By applying this framework, they were able to more accurately predict the outcomes of melanoma patients treated with first-line anti-PD1 or anti-CTLA4 than if they had used other biomarkers such as PD-L1 level and mutation load [13].

The advancements in biomarker technologies for the identification, the validation, and the multiplexing of genomic and proteomic signatures may provide new knowledge to enable researchers to develop immunotherapy response prediction markers. The lack of consensus on standardized approaches, as well as the difficulty of designing clinical trials with sufficient sample sizes and power, are currently the main constraints in developing optimal biomarker panel selection for effective personalized medicine [14].

Future success in cancer treatment depends on effective combinations of methods. Surgery, chemotherapy, radiation, and targeted drugs all directly attack the cancer cell. In fact, we must consider that a tumor is much more than an ensemble of cancer cells; it is a complex environment in which other cell types have key functions and, sometimes, corrupt roles. Immunotherapy, instead of focusing on cancer cells, targets immune cells. Each of these therapies functions differently, but when researchers identify the best ways to combine them, it could enable the weakening of whole tumors and prepare immune systems to permanently eradicate cancer.

This editorial is not a summary of evidence and outstanding studies, but aims to provide knowledge of new translational research studies and tools in the vast field of clinical and experimental immunotherapy. It intends to pave the way for new therapeutic options and their combinations, as well as biomarker investigation technologies and algorithms, to study the mechanisms of response and resistance in the field of immunotherapy.
